# Impacts of Chromatin States and Long-Range Genomic Segments on Aging and DNA Methylation

**DOI:** 10.1371/journal.pone.0128517

**Published:** 2015-06-19

**Authors:** Dan Sun, Soojin V. Yi

**Affiliations:** School of Biology, Georgia Institute of Technology, Atlanta, GA, 30332, United States of America; Harbin Medical University, CHINA

## Abstract

Understanding the fundamental dynamics of epigenome variation during normal aging is critical for elucidating key epigenetic alterations that affect development, cell differentiation and diseases. Advances in the field of aging and DNA methylation strongly support the aging epigenetic drift model. Although this model aligns with previous studies, the role of other epigenetic marks, such as histone modification, as well as the impact of sampling specific CpGs, must be evaluated. Ultimately, it is crucial to investigate how *all* CpGs in the human genome change their methylation with aging in their specific genomic and epigenomic contexts. Here, we analyze whole genome bisulfite sequencing DNA methylation maps of brain frontal cortex from individuals of diverse ages. Comparisons with blood data reveal tissue-specific patterns of epigenetic drift. By integrating chromatin state information, divergent degrees and directions of aging-associated methylation in different genomic regions are revealed. Whole genome bisulfite sequencing data also open a new door to investigate whether adjacent CpG sites exhibit coordinated DNA methylation changes with aging. We identified significant ‘aging-segments’, which are clusters of nearby CpGs that respond to aging by similar DNA methylation changes. These segments not only capture previously identified aging-CpGs but also include specific functional categories of genes with implications on epigenetic regulation of aging. For example, genes associated with development are highly enriched in positive aging segments, which are gradually hyper-methylated with aging. On the other hand, regions that are gradually hypo-methylated with aging (‘negative aging segments’) in the brain harbor genes involved in metabolism and protein ubiquitination. Given the importance of protein ubiquitination in proteome homeostasis of aging brains and neurodegenerative disorders, our finding suggests the significance of epigenetic regulation of this posttranslational modification pathway in the aging brain. Utilizing aging segments rather than individual CpGs will provide more comprehensive genomic and epigenomic contexts to understand the intricate associations between genomic neighborhoods and developmental and aging processes. These results complement the aging epigenetic drift model and provide new insights.

## Introduction

Recent advances in DNA methylation analysis technology and the availability of large aging cohorts have enabled dramatic progression in our knowledge regarding DNA methylation changes with aging. Recent large-scale studies have clearly demonstrated that DNA methylation patterns diverge, or undergo ‘epigenetic drift’, with aging. In particular, aging-associated global hypo-DNA methylation has been observed across several tissues and cell types [1–4]. On the other hand, promoters and CpG islands tend to exhibit hyper-methylation with aging as exceptions to this global pattern [2,5–12]. Moreover, other epigenetic marks also exhibit aging-associated changes often in conjunction with DNA methylation. For example, CpGs are hyper-methylated in polycomb target genes and bivalent chromatin domains [13–16], whereas CpGs in enhancers often exhibit aging-associated hypo-methylation [11,15–17]. Another exciting discovery involves the identification of ‘aging CpGs’, which can be used to estimate ‘biological’ ages of specific individuals [15,18–22].

Despite these advancements, several fundamental questions remain. A prominent issue is the potential bias introduced by non-random sampling of CpGs. Most previous studies employed a sampling strategy to reduce the number of CpGs from ~30 million (the total number of CpGs in the human genome) to statistically manageable numbers. For instance, the widely used Illumina 27K Chip analyzes approximately 0.1% of total CpGs in the human genome. These ‘selected’ CpGs, especially those used in commercially developed methylation arrays, are often biased toward promoters and CpG islands. However, DNA methylation is also highly prevalent in gene bodies and distal intergenic regions, with significant functional consequences (e.g., [23–26]). Moreover, most CpGs that exhibit variation of DNA methylation are located in gene bodies and intergenic regions [27]. The next-generation methylation chip (e.g., Illumina 450K Chip) examines ~1.5% of total CpGs in the human genome, with similarly biased distributions favoring promoters and CpG islands [27]. Thus, sampling strategies could have significant consequences on the inference of DNA methylation changes with aging. Another important potential factor involves the variability of aging-associated DNA methylation changes across cell types and tissues. Although common aging modules may exist across different tissues (e.g., [5,11,15,28,29]), the extent to which tissue- or cell type-specific processes drive aging-associated DNA methylation changes remains unknown [29].

To shed light on these questions, it is necessary to compare patterns of aging-associated DNA methylation among different tissues. Moreover, performing such analyses using data from whole genome bisulfite sequencing, thus in principle examining *all* CpGs in the human genome, will yield *unbiased* genome-wide patterns. In addition, given that previous studies on the association between aging CpGs and chromatin states typically relied on limited numbers of *a priori* selected CpGs, it is useful to re-evaluate the relationship between chromatin states and CpG methylation using bisulfite-sequencing data.

Here, we perform a comprehensive analysis of DNA methylation variation with aging using recently generated whole genome bisulfite sequencing DNA methylation data from the frontal cortex brain region of eight individuals [25,30]. We also generate a chromatin state map of the frontal cortex utilizing extensive histone modification data from the NIH RoadMap Epigenomics Project [31]. Although various patterns that are consistent with the random ‘aging epigenetic drift’ model are identified, we also observe that specific genomic regions follow distinctive aging patterns of DNA methylation. Notably, the integration of DNA methylation data sets and chromatin state maps reveals extensive co-variation of these two epigenetic marks. By comparing these results with previously reported genome-wide DNA methylation variation from CD4+ T lymphocytes [2], we can begin to address the heterogeneity of aging patterns among tissues. Furthermore, we introduce a new method to identify ‘aging segments’. Aging segments are genomic regions with consecutive CpGs whose methylation changes in a concerted fashion with aging. Analyses of aging segments provide insights into the co-variation between DNA methylation and chromatin states as well as differences in the molecular mechanisms of aging-associated hyper- and hypo-DNA methylation.

## Results

### Tissue-divergent patterns of epigenetic drift based on nucleotide resolution whole-genome methylation maps

**Fig 1:**
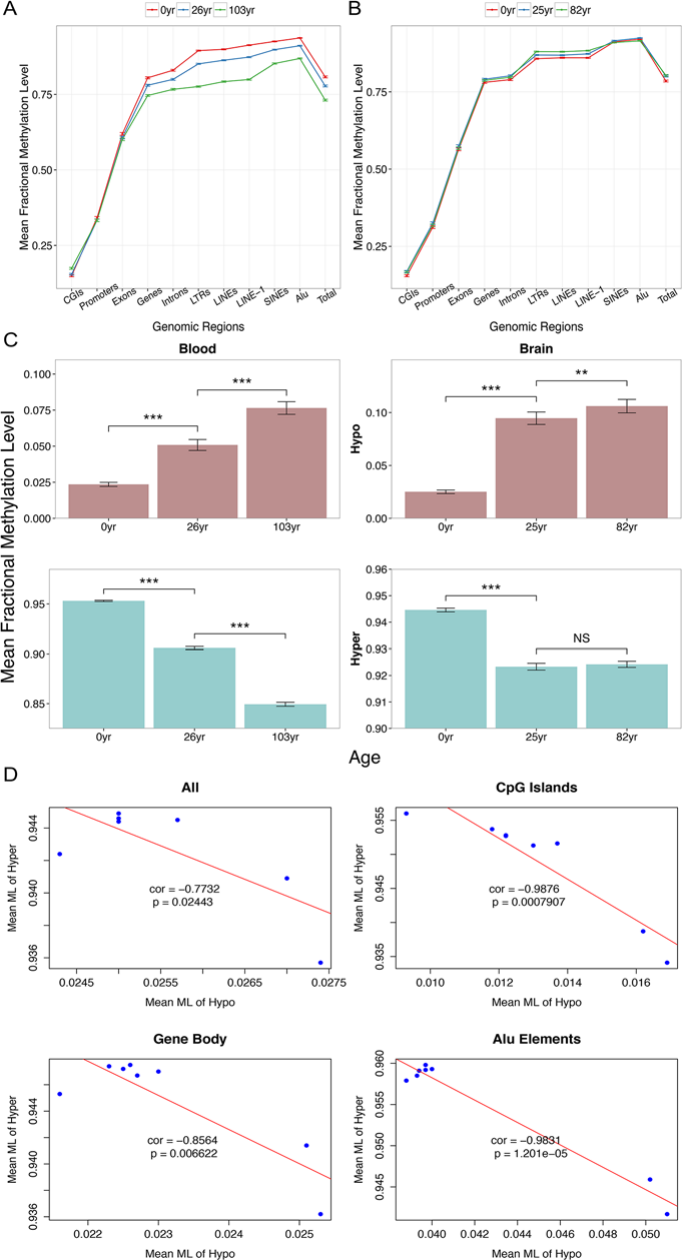
Aging-associated changes in DNA methylation based on whole-genome bisulfite sequencing data. To represent aging patterns more clearly and in a comparable manner between the two data sets, only brain data from three individuals (with comparable ages to those in the blood data set) are presented. Patterns from all eight individuals are highly similar to the simplified pictures. Data from 10,000 randomly selected CpGs are presented. (A) Comparisons of mean fractional methylation levels among 3 individuals (with 95% confidence intervals) across different genomic regions in blood. (B) Comparisons of mean fractional methylation levels (with 95% confidence intervals) among 3 individuals across different genomic regions in brain. (C) Data from extremely hypo-methylated (fractional methylation levels < 0.2) CpGs (upper panel) and extremely hyper-methylated (fractional methylation levels > 0.8) CpGs from the two data sets. (D) Methylation levels of extremely hyper- and hypo-methylated CpGs are strongly negatively correlated in brain.

We first describe global patterns of DNA methylation with respect to aging using whole-genome bisulfite sequencing data. Of the total 26.8 X 10^6^ autosomal CpG sites (in the human genome hg19 / GRCh37 build), we examine 25.4 X 10^6^ sites in frontal cortex samples from eight individuals ranging in age from newborn to 82 years and 9.0 X 10^6^ sites in CD4+ T-cells (blood) samples from three individuals (newborn and 26 and 103 years old). These comprehensive data confirm that the whole genome is heavily methylated: the average fractional methylation levels are 0.7976 (± 0.0093)/CpG in brain and 0.7756 (± 0.0097)/CpG in blood. Patterns of DNA methylation variation across different functional regions are generally consistent with previous findings: promoters, gene bodies and repetitive regions exhibit low, medium and high methylation, respectively (Fig 1A and 1B). CpG islands and *Alu* elements exhibit the lowest and highest levels of DNA methylation, respectively, in both data sets (Fig 1A and 1B).

With respect to genome-wide patterns of aging-associated DNA methylation changes, the blood data unequivocally indicate global hypo-methylation with aging accompanied with local hyper-methylation with aging at CpG islands (Fig 1A, S1A Fig). These patterns are hallmarks of the global aging epigenetic drift model [1–3,9,10,14,21,29,32,33]. Interestingly, brain methylation maps reveal a very different picture. Compared with the blood data set, the brain samples exhibit much less variation of DNA methylation with aging (Fig 1B, S1B Fig). Moreover, contrary to the aging epigenetic drift model, a slight increase in DNA methylation is noted in most genomic regions of brain.

However, although the global pattern in the brain data does not conform to the previous model, CpG sites with extreme initial DNA methylation follow the expected trend. We examined extremely hyper-methylated (fractional methylation level > 0.8) and hypo-methylated (fractional methylation level < 0.2) CpGs. A significant increase in DNA methylation with age in hypo-methylated CpGs and a significant decrease in DNA methylation in hyper-methylated CpGs are detected regardless of the genomic context and tissue type. These patterns are especially pronounced during the time period up to young adulthood (ages 26 and 25 in blood and brain data, respectively) (Fig 1C, S2 Fig, paired *t*-test, one-tailed). Notably, no statistically significant differences in brain data were observed in samples from individuals between the ages 25 and 82, whereas the blood data exhibit a significant decrease in DNA methylation. Nevertheless, mean DNA methylation levels of hyper-methylated and hypo-methylated CpGs are highly negatively correlated in brain data, and this correlation is most pronounced in CpG islands (Fig 1D). These analyses complement the aging epigenetic drift model by supporting its predictions from extremely hyper- and hypo-methylated CpGs in brain.

### Distinctive patterns of aging DNA methylation across chromatin states in brain

**Fig 2:**
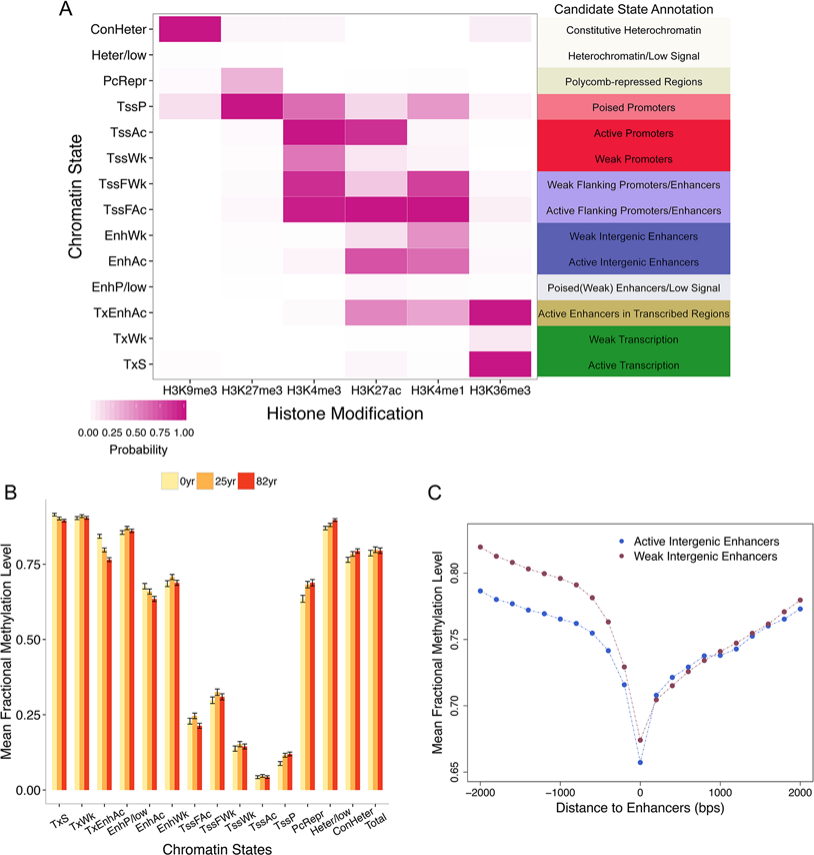
Chromatin states and DNA methylation in brain data. (A) The emission probability matrix indicating the composition of 6 histone modifications in each state. Candidate annotation for each chromatin state is also presented. (B) DNA methylation levels of CpGs located in different chromatin states. Data (mean fractional methylation levels ± standard error) from three individuals (a newborn as well as a 25-year-old and 82-year-old individual) are presented. In total, 1,000 CpGs were randomly chosen for each state. (C) Enhancers residing in distal intergenic regions (states *EnhWk* and *EnhAc*) are significantly hypo-methylated compared with nearby regions. The position 0 indicates the focal enhancer, and the levels of DNA methylation up to 2 kb from the enhancers in either direction are presented (200-bp bin size).

We then sought to elucidate detailed patterns of coordinated age-associated changes between DNA methylation and chromatin states. Here, we focus on brain data, utilizing the availability of eight relatively well-separated aging samples. We first generated a brain-specific chromatin state map. From the 6 different histone modification profiles (H3K9me3, H3K27me3, H3K27Ac, H3K4me1, H3K4me3 and H3K36me3) generated by Chip-Seq of brain tissue from the NIH Roadmap Epigenomics database, we inferred 14 chromatin states using ChromHMM [34,35] (Materials and Methods, S1 Text, S3 Fig). These include the following defined states (Fig 2A): active transcription (*TxS*), weak transcription (*TxWk*), active enhancers in transcribed regions (*TxEnhAc*), poised/weak enhancers or low signal (*EnhP/low*), active intergenic enhancers (*EnhAc*), weak intergenic enhancers (*EnhWk*), active 5’ flanking promoters/enhancers (*TssFAc*), weak 5’ flanking promoters/enhancers (*TssFWk*), weak promoters (*TssWk*), active promoters (*TssAc*), poised promoters (*TssP*), polycomb-repressed regions (*PcRepr*), heterochromatin or low signal (*Heter/low*), and constitutive heterochromatin (*ConHeter*). The proportion of sites assigned to different chromatin states is presented in S4 Fig.

We then examined variations in DNA methylation across different chromatin states. DNA methylation levels of different chromatin states are highly and significantly different from each other (Fig 2B). Promoters, in particular active promoters (*TssAc*), exhibit the lowest methylation (0.0408 ± 0.0041). On the other hand, transcription (*TxS*, *TxWk*) and heterochromatin or low signal (*Heter/low*) states exhibit the highest mean methylation levels (Fig 2B). These results are consistent with strong gene body methylation (*TxS* and *TxWk*) and methylation of heterochromatic regions (*Herer/low*). Interestingly, active and weak enhancers that are distally located in intergenic regions (*EnhAc*, *EnhWk*) are also highly methylated (65–70% methylated), which is in contrast to previous results reporting that enhancers are generally hypo-methylated (e.g., [36]). However, the DNA methylation levels of these regions are highly significantly reduced compared with flanking intergenic regions (Fig 2C), which is concordant with the relative hypo-methylation of enhancers [36,37]. On the other hand, states harboring strong chromatin signatures enhancers (including H3K4me1 and H3K27Ac) and located nearby transcription start site (states *TssFAc*, *TssFWk*, annotated as flanking promoters/enhancers) are strongly hypo-methylated.

**Fig 3:**
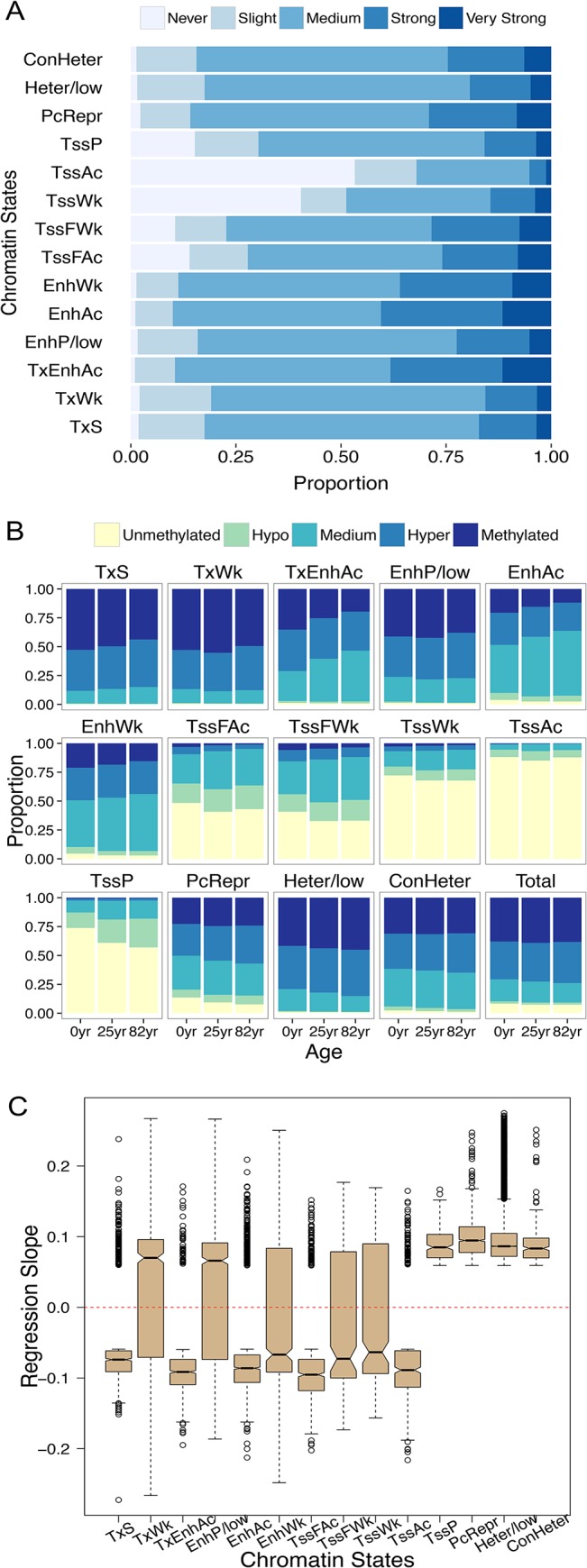
DNA methylation changes in CpGs across the 14 chromatin states. To facilitate the visualization of aging-associated DNA methylation changes, we divided CpGs into five classes based on their regression coefficients from the linear model. CpGs that rarely exhibit DNA methylation alterations (absolute value of regression coefficients < 0.0002, 5% quantile), those slightly exhibit DNA methylation alterations with aging (absolute regression coefficients in [0.0002, 0.0034), 5~20% quantiles), CpGs that change DNA methylation medially (absolute regression coefficients between [0.0034, 0.0272), 20~80% quantiles), and CpGs that strongly (absolute regression coefficients between [0.0272, 0.0591), 80~95% quantile) and very strongly (absolute regression coefficients > = 0.0591, top 5%) alter DNA methylation are referred to as never (never), slightly (slight), medially (medium), strongly (strong) or very strongly (very strong), respectively. CpGs with fractional methylation levels between 0 ~ 0.05 and 0.05 ~ 0.2 are defined as unmethylated and hypo-methylated, respectively. CpGs with fractional methylation levels between 0.95 ~ 1.00 and 0.80 ~ 0.95 are defined as methylated and hyper-methylated, respectively. (A) The proportions of CpGs in different classes in genomic regions occupied by different chromatin states. (B) Changes of CpG methylation with aging across three individuals (newborn, 25 years old and 82 years old). (C) The mean regression slopes of the most significant (top 5% in regression coefficients, *P*-values < 10^−3^) aging CpGs reveal contrasting patterns of aging-associated DNA methylation changes between active versus poised/repressed chromatin states.

Moreover, different chromatin states exhibit different degrees of aging-associated methylation changes (Fig 3A). A linear regression model was employed using ages as predictors and DNA methylation levels as response (Materials and Methods, S5 Fig). Combining chromatin states and DNA methylation changes with aging, we demonstrate that CpGs that are located in active promoters (*TssAc*) tend to remain stably hypo-methylated throughout the aging process and exhibit the least amount of variation (Fig 3A and 3B). On the other hand, active enhancers located in intergenic regions and gene bodies (*TxEnhAc* and *EnhAc*) exhibit the most dynamic patterns of DNA methylation during aging, undergoing significant hypo-methylation with aging. Interestingly, chromatin states harboring enhancer signals yet residing nearby TSSs (such as *TssFWk* and *TssFAc*, Fig 2A) exhibit less variability with aging (Fig 3A and 3B). In contrast, CpGs located in the ‘poised promoters’ state (*TssP*) and polycomb-repressed regions (*PcRepr*) exhibit substantial hyper-methylation with aging (Fig 3B). Overall, regions corresponding to chromatin states associated with active histone marks tend to exhibit negative DNA methylation changes with age (Fig 3C). In contrast, DNA methylation of chromatin states that harbor repressive or poised histone marks are positively correlated with age (Fig 3C).

One caveat of the present analysis is that it utilized the chromatin state map generated from data from a specific individual. Consequently, the observed pattern may reflect individual-specific patterns. To take into account such variability, we first extracted genomic regions that exhibit consistent chromatin states in 9 different cell lines (including embryonic stem cells, erythrocytic leukemia cells, B-lymphoblastoid cells, hepatocellular carcinoma cells, umbilical vein endothelial cells, skeletal muscle myoblasts, normal lung fibroblasts, normal epidermal keratinocytes and mammary epithelial cells) [35]. We subsequently re-examined the relationship between chromatin states and DNA methylation in this ‘consistent’ chromatin state map. Interestingly, intergenic distal enhancers (states 5 and 7 in ref [35]) tend to variable across the 9 cell lines. This observation is potentially explained by the highly variable epigenetic nature of enhancers across various biological processes [30,38–41]. Genomic regions corresponding to other chromatin states across the 9 cell lines reveal similar aging DNA methylation dynamics as shown above (S6 Fig).

### Novel aging segments define distinctive epigenomic and functional neighborhoods

Neighboring positions in the genome may exhibit similar epigenetic profiles and facilitate complex regulation [42,43]. For example, DNA methylation levels of nearby sites are highly correlated in diverse genomes [44–46]. Consequently, it is of great interest to investigate whether explicit genomic neighborhoods exist that epigenetically respond to aging in a similar manner. Aging methylation maps of approximately all CpGs in the human genome provide an exciting opportunity to explore this question. Specifically, we examine clusters of adjacent CpGs that exhibit similar patterns of methylation changes with aging using the maximal scoring subsequence algorithm [47]. This approach aims to identify all non-overlapping and continuous subsequences with highest local scores and is used in a variety of genomic analyses (e.g., [48–50]). In our approach, subsequences correspond to clusters of adjacent CpG that exhibit similar positive or negative correlations with age (Materials and Methods). We subsequently identified 133,650 positive aging segments (length: 140.2 X 10^6^ bps) and 7,661 negative aging segments (length: 31.9 X 10^6^ bps) from the brain data (S7 Fig, **S1 Table**).

This novel analysis reveals several intriguing aspects of the epigenomic response to aging. First, genomic regions covered by the positive and negative aging segments exhibit variable lengths in the brain data (S7 Fig). Although the number of positive individual CpGs (those that increase methylation with aging) is highly similar to the number of negative CpGs based on the regression analysis (50.9% versus 45.7%, not including zeros; S5 Fig), the total length of negative aging segments is considerably shorter than positive segments. This observation, at least on the surface level, is consistent with the notion that increases in DNA methylation reflect regulation, whereas decreases in DNA methylation are caused by stochastic processes (e.g., [17,29]).

**Fig 4:**
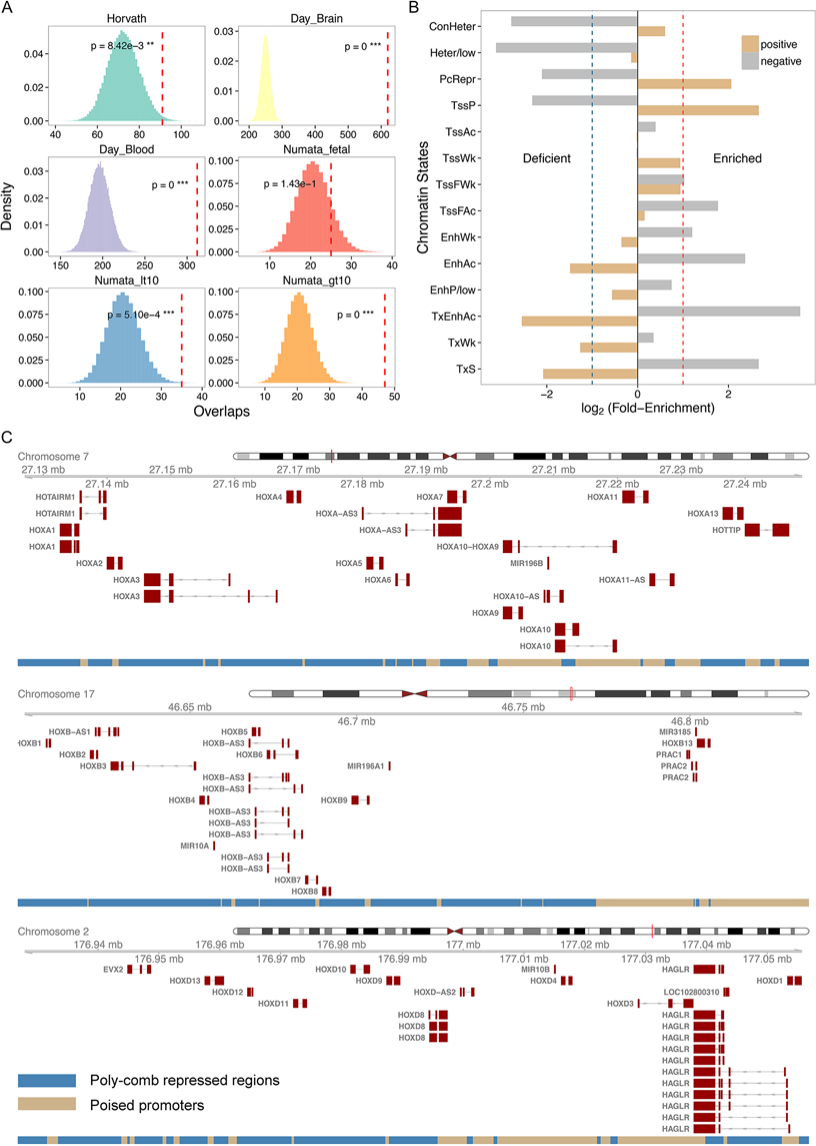
Aging segment analyses. (A) Significant overlaps between aging segments and previously identified ‘aging CpGs’. Each panel represents the distribution of the expected overlaps between aging segments and aging CpGs based upon random permutation. The observed overlap is denoted by a red dotted line, and its probability (based upon the permutation test) is indicated based on aging CpGs identified in Horvath [15]; Day et al. [52] using aging CpGs from brain and blood; and Numata et al. [51] using aging CpGs from fetal brains as well as brains from patients less than or greater than 10 years of age. (B) Enrichment and deficiency of different chromatin states in positive and negative aging segments. (C) Three HOX gene clusters in positive aging segments are occupied by polycomb-repressed regions and poised promoters.

We next examine the overlaps between the aging segments defined in this study using the aging CpGs identified in previous studies [11,15,51]. A critical difference between these previous studies and the current study is that the previous studies utilized methylation arrays, which include CpGs that were pre-selected (Illumina Infinium 27K chip, approximately 27,000 CpG sites). On average, the Infinium 27K BeadChip covers 2 CpG sites per RefSeq gene, and this value is several orders of magnitude fewer CpG sites than that analyzed in the current study. On the other hand, these studies exhibit greater statistical power than our study because they focus on fewer positions (reducing the burden of multiple testing problem) in a considerably larger number of samples. Despite such differences, we observe that previously reported aging CpGs are highly enriched in aging segments (Fig 4A). In other words, our aging segments effectively capture previously identified aging patterns. Interestingly, positive aging segments exhibit enhanced enrichment for previously identified aging CpGs compared with negative segments (S8A and S8B Fig); the only exceptions include fetal and child brain samples [51]. This is again consistent with the idea that positive aging segments more strongly reflect regulatory processes compared to negative aging segments.

Positive and negative aging segments also exhibit distinctive patterns of enrichment and deficiencies of specific chromatin states. Negative aging segments are enriched for strong transcription, active intergenic enhancers and 5’ flanking regions (*TxS*, *TxEnhAc*, *EnhAc*, *TssFAc*). In addition, these segments are significantly devoid of poised promoters, polycomb-repressed regions and repetitive regions (*ConHeter*, *Heter*/*low*, *PcRepr*, *Tssp*) (Fig 4B). In contrast, positive aging segments are enriched for poised promoters and polycomb-repressed regions but lack transcribed regions and active enhancers (Fig 4B). These observations are consistent with results from the previous section, indicating coordinated epigenetic responses between DNA methylation and chromatin states. Furthermore, positive and negative aging segments are enriched in distinctive gene functional categories (Table 1). Positive aging segments are involved in cell adhesion and development. In contrast, negative aging segments harbor many genes involved in RNA processing, metabolic processes, and protein ubiquitination (Table 1).

**Table 1:** Gene ontology (GO) enrichments in positive and negative aging segments of brain. *q*-values are *P*-values and are adjusted via Bonferroni correction.

	GO Term	Fold Enrichment	q-value
Positive	Cell adhesion	1.3	2.80E-10
	Embryonic morphogenesis	1.4	3.40E-07
	Skeletal system development	1.4	2.00E-06
	Homophilic cell adhesion	1.6	2.80E-06
	Cell-cell signaling	1.3	8.50E-06
	Pattern specification process	1.4	1.30E-05
	Regionalization	1.5	1.80E-05
	Cell-cell adhesion	1.4	4.70E-05
	Ion transport	1.2	8.10E-05
	Anterior/posterior pattern formation	1.5	2.10E-04
Negative	RNA splicing	2.4	4.60E-07
	RNA processing	2.0	5.50E-07
	Regulation of ubiquitin-protein ligase activity	4.1	1.00E-06
	Regulation of ligase activity	3.9	2.60E-06
	mRNA metabolic process	2.1	1.00E-05
	Positive regulation of ubiquitin-protein ligase activity	4.0	1.40E-05
	Regulation of protein ubiquitination	3.4	1.90E-05
	Positive regulation of protein ubiquitination	3.6	3.00E-05
	Positive regulation of ligase activity	3.9	3.40E-05
	Positive regulation of ubiquitin-protein ligase activity during mitotic cell cycle	4.0	4.00E-05

### Common and divergent patterns between blood and brain based on whole genome data

To identify common aging patterns across tissues, we analyzed blood data (CD4+ T cells from three individuals, Heyn et al. [2]). Based on the extensive 51 chromatin state map of CD4+ T cells from Ernst and Kellis [53], we determined whether different chromatin states exhibit differential patterns of DNA methylation with aging. Unlike the brain data set (S9A Fig), most CpGs in the blood data set exhibit hypo-methylation with aging across different chromatin states (S9B Fig), consistent with the observed genome-wide hypo-methylation with aging (Fig 1A). Of note, the weak/repressed promoters exhibit hyper-methylation with aging (positive regression coefficients with age) in the blood data as well (S9B Fig).

When we applied the maximal segment algorithm, the number of resulting negative aging segments is significantly greater than the resulting positive aging segments (36,294 negative segments vs. 2,845 positive segments, S7 Fig, S1 Table). This finding is also consistent with that the blood data set exhibits pervasive hypo-methylation with aging (Fig 1). However, these results should be taken with caution, as they are exclusively based on three individuals. A direct comparison of the brain and blood aging segments is technically not feasible due to the differences in the sample size (eight versus three individuals) as well as the total numbers of CpGs analyzed (three-fold difference between the two data sets, Materials and Methods). Nevertheless, 1,174 and 1,512 genes are included in the positive and negative segments, respectively, in both samples (S3 Table). Interestingly, genes appearing on the positive segments in both samples are highly over-represented in gene ontology terms associated with development (Table 2). For example, genes corresponding to the term ‘embryonic morphogenesis’ are 5-fold enriched in the positive aging segments. This term includes genes such as HOX genes, which are epigenetically suppressed post-embryonically. Common positive aging segments thus capture genomic neighborhoods of genes that are epigenetically silenced via gradual increases in DNA methylation. Genes located in the negative aging segments in both samples are enriched in GO terms related to metabolic process and phosphorylation (Table 2).

**Table 2:** Genes located in common aging segments in brain and blood exhibit distinctive functional enrichments. *q*-values are *P*-values are adjusted via Bonferroni correction.

	GO Term	Fold Enrichment	q-value
Positive	Pattern Specification Process	5.2	6.20E-17
	Embryonic Morphogenesis	4.7	5.90E-16
	Regionalization	5.5	2.60E-13
	Anterior/Posterior Pattern Formation	6.1	4.40E-11
	Neuron Differentiation	3.3	1.10E-09
	Homophilic Cell Adhesion	5.9	3.50E-09
	Tube Development	4.4	6.00E-09
	Skeletal System Development	3.7	6.50E-09
	Appendage Development	6.6	7.20E-09
	Limb Development	6.6	7.20E-09
Negative	Phosphorus Metabolic Process	1.8	1.90E-02
	Phosphate Metabolic Process	1.8	1.90E-02
	Transmission of Nerve Impulse	2.5	3.50E-02
	Protein Amino Acid Phosphorylation	2	4.80E-02
	Phosphorylation	1.9	4.90E-02

## Discussion

Our study demonstrates widespread aging-associated variations in DNA methylation in brain, by examining approximately all CpGs in the human genome. Understanding the molecular mechanisms of such epigenetic drift will advance our knowledge on aging and aid in elucidating the dynamics of DNA methylation turnover at individual CpG sites. One previously observed pattern in the aging process and cancer involves global hypo-methylation coupled with promoter hyper-methylation [1,2,29,54]. After re-examining this hypothesis using whole-genome methylation maps, the expected pattern is observed for blood but not for brain (Fig 1A and 1B). In light of these results, we propose that CpGs with extreme initial methylation levels are more prone to DNA methylation drift regardless of genomic context (Fig 1C and 1D). Age-associated dysregulation of DNA methylation maintenance may cause these extreme states of hypo- and hyper-methylation to revert to intermediate methylation levels.

However, for the remaining genomic regions, the direction of methylation changes with aging cannot be exclusively explained based upon the initial or mean methylation levels. For example, many hypo-methylated promoters exhibit minimal epigenetic drift with aging. Instead, we demonstrate that integrating histone modification data with aging DNA methylation maps provide the specific chromatin context to the observed patterns in brain (Fig 2 and Fig 3).

Among the 14 chromatin states defined, active intergenic enhancers exhibit the most dynamic variation in DNA methylation with aging (Fig 3). This finding is consistent with previous studies indicating that enhancer hypo-methylation occurs with aging [11,13–17], thus emphasizing the co-variation of histone modification and DNA methylation marks in aging. Furthermore, given that we could examine a much larger number of CpGs compared with previous studies, we demonstrate that originally hyper-methylated intergenic and intragenic enhancers are subject to strong hypo-methylation, which differs from the patterns for proximal enhancers. This observation may provide clues to identifying the underlying mechanisms of the co-variation between chromatin states and DNA methylation changes with aging. For example, many intergenic and intragenic enhancers are located in low CpG density genomic regions with high initial DNA methylation, whereas proximal enhancers and promoters are typically located in regions of high CpG density with low initial DNA methylation. These genomic differences and the initial epigenetic signals may affect how DNA methylation levels of specific CpGs change with aging. Future studies are necessary to refine the co-variation between chromatin states and DNA methylation changes, and to elucidate the underlying mechanisms. We also demonstrate that poised promoters and polycomb-repressed regions continue to increase DNA methylation with aging. A prominent example of this phenomenon is observed in the HOX clusters, which are epigenetically suppressed cooperatively via both DNA methylation and histone modifications (Fig 4C). Hyper-methylation of poised promoters could also be related to the initiation of *de novo* DNA methylation following H3K27me3 modification induced by polycomb complexes [55,56].

Comparing aging whole-genome methylation maps of brains to those of blood reveals intriguing similarities and differences between the two tissues. In both data sets, age-associated changes in DNA methylation are concentrated in intragenic and intergenic regions instead of promoters or exons (Fig 1A and 1B). Unfortunately, commercially available methylation chips tend to target genic and promoter regions, thus potentially limiting our ability to grasp the full extent of DNA methylation changes with aging. Numerous previous analyses of DNA methylation and aging focused on CpG islands and consequently observed aging-associated hyper-methylation [57–60]. We also identify some common features of co-variation between chromatin states and DNA methylation variation. Specifically, aging-associated hyper-methylation of poised promoters and hypo-methylation of distal/intergenic enhancers are apparent in both data sets.

Interestingly, blood samples exhibit a pronounced global hypo-methylation with aging [2], whereas brain samples do not exhibit an obvious pattern at the global level (Fig 1). Although caution should be used when interpreting this difference given the small number of samples, blood samples notably exhibited consistent hypo-methylation across many different types of CpGs compared with other tissues in a previous study [5]. Additional aging whole-genome methylation maps from diverse tissues will elucidate the details of the long-observed tissue differences in aging patterns noted among tissues. For example, epidermal whole-genome methylation maps exhibit minimal differences between young versus old populations; however, only two whole-genome methylation maps were compared [16]. The causes of among tissue differences in aging patterns are unclear. Some suggest the differences in proliferative potential across tissues as a factor in these differences [61]. This hypothesis may be worth re-visiting in light of a recent suggestion that a similar factor underlie differential cancer susceptibility across tissues [62].

Although analysis of whole-genome nucleotide maps provides an unbiased representation of aging and DNA methylation, it also offers a significant challenge to commonly used linear model methods given the extremely large number of CpGs in the entire genome, thus posing a tremendous burden of multiple testing corrections. Additionally, a strong spatial correlation of DNA methylation of nearby CpGs has been observed across diverse species [44–46]. To overcome such statistical limitations and efficiently utilize the spatial correlation, here we investigated clusters of CpGs that respond to aging in a similar manner. Our method offers a statistically robust framework to analyze aging whole-genome methylation maps. Notably, aging CpGs identified in previous studies using Illumina 27K data [15,51,52] are highly significantly enriched in our aging segments, suggesting that the aging segments capture biologically meaningful genomic neighborhoods. Moreover, aging segments could be more robust in the presence of SNPs. Aging CpGs are highly affected by individual SNPs occurring at each CpGs, whereas aging segments harbor a large number of CpGs.

We also observe intriguing functional ontology associations with positive and negative aging segments. Positive aging segments, which exhibit gradual hyper-methylation with aging, are highly enriched with genes associated with developmental ontology terms in the brain (Table 1). This finding is consistent with the notion that DNA methylation down-regulates neurodevelopmental genes [25]. Notably, positive aging segments are highly enriched for the Homeobox domain in the DAVID INTERPRO database (*q* < 10^−13^ after Bonferroni correction). In particular, three HOX gene clusters (A, B and D) reside in positive aging segments (Fig 4C) occupied by poised promoters (*TssP*) and polycomb-repressed regions (*PcRepr*). These results indicate that DNA methylation and histone modification synergistically suppress HOX expression in adult brains [25,63].

Negative aging segments in brain are enriched with genes associated with metabolism, RNA processing, and protein ubiquitination (Table 1). Enrichment of these gene ontology terms in the negative aging segments may indicate epigenetic up-regulations of these genes. For example, protein ubiquitination is an essential posttranslational modification for the removal of damaged or misfolded proteins [64]. Thus, ubiquitin plays a critical role in proteome homeostasis during aging [65,66]. Impairment of ubiquitination pathways leads to the accumulation of damaged and aggregated proteins, which are associated with aging as well as neurodegenerative disorders, such as Alzheimer’s [67,68]. Gradual hypo-methylation of genes in the protein ubiquitination pathways may indicate an epigenetic up-regulation of this pathway during aging.

Our study has several potential caveats. The brain data used only included 8 individuals, and the blood data were derived from 3 individuals. In addition, the fact that data from brain and blood were obtained from different sets of individuals should be taken into consideration when comparing the extent of epigenetic drift between the two tissues. The histone modification data were obtained from a single individual; however, these data were complemented with data from multiple cell types. In addition, the brain methylation maps were generated from cortex samples and thus could be affected by cellular heterogeneity [69]. A recent study suggests that cellular heterogeneity may have only negligible effects on aging-associated DNA methylation changes [15]. Nevertheless, our analyses provide a good comparison with previously identified aging patterns from similar cortex samples [11,15,28,51,57]. Analyses of DNA methylation and chromatin modification data from a larger number of biological replicates obtained from cell-sorted samples will allow researchers to avoid the aforementioned potential biases. Such data will almost certainly become available in a near future. Our methods will be fully applicable to such data and help reveal the details of genome-wide differences across tissues and cell types and ultimately elucidate the molecular mechanisms underlying such differences and similarities between tissues.

## Materials and Methods

### DNA methylation and gene expression data

We analyzed DNA methylation maps generated by whole-genome bisulfite sequencing from the frontal cortex [25,30] samples from eight individuals spanning a diverse spectrum of ages (a 35-day-old male; 2-, 5-, 12-, 16- and 25-year-old males; 81- and 82-year-old females). We also analyzed whole-genome methylation maps of CD+ T-cells from a male newborn, a 26-year-old individual of unknown sex and a 103-year-old male [2]. In total, 25.4 X 10^6^ CpGs from frontal cortex and 9.0 X 10^6^ CpGs from CD4+ T-cells were analyzed. To eliminate confounding effects of gender [51,70], data from sex chromosomes were excluded. The fractional methylation level of each CpG was calculated as “the number of methylated reads / total number of reads (= number of methylated reads + number of unmethylated reads)” [37,70].

Our main focus was the first data set (‘brain’ data set). We limited our interpretation of the second data set (‘blood’ data set) as it contained only three samples and fewer mapped CpGs. Gene expression data were obtained from the BrainSpan Atlas of the Developing Human Brain [63,71].

### Age-based methylation modeling

Age-associated DNA methylation changes at individual sites were assessed using a linear model [11,15,51]. We used *ln*(*age*+1) as a predictor and fractional methylation level as a response variable to account for the rapid changes of DNA methylation that occur during early development [15,51]. Regression coefficients from this model indicate the strength and direction of age-associated DNA methylation changes.

### Chromatin states map of brain

Chip-seq data containing 6 chromatin modifications H3K9me3, H3K27me3, H3K27Ac, H3K4me1, H3K4me3 and H3K36me3 from the prefrontal cortex of a 75-year-old female were downloaded from NIH Roadmap Epigenomics (www.roadmapepigenomics.org/). The GSM numbers for these data sets are GSM772833 for H3K27me3, GSM772834 for H3K9me3, GSM773012 for H3K4me3, GSM773013 for H3K36me3, GSM773014 for H3K4me1, GSM773015 for H3K27ac, and GSM773010 for ChIP-Seq input. We used ChromHMM [34] to train a multivariate Hidden Markov Model. First, histone modification reads were transformed into binary values using a default 200-bp bin size. Second, the LearnModel function was used to learn models. A transmission probability matrix and an emission probability were generated. Based on these priors, each bin was given posterior probabilities for each state. The state with the highest probability was used to label that bin. To maximally define possible chromatin states with 6 histone modifications, 7 to 15 state models were trained. We selected the 14 state model because it demonstrated key interactions amongst the chromatin marks without incurring unnecessary redundancies.

### Identifying aging segments

We used a maximal scoring subsequence algorithm [47] to define aging segments. This approach aims to identify all non-overlapping and continuous subsequences with maximal local scores [47,48]. For all mapped CpGs, the *t-*statistics from the linear regression model were used as pre-scores. It is advantageous to use *t*-statistics from the regression because these values represent the impacts of both the strength of correlation (*P*-value) as well as the degree of the changes with aging (regression coefficients). After excluding outliers, the ‘pre-scores’ were then normalized to a [-1,1] scale.

Each mapped CpG was given a positive score for increase of DNA methylation with aging or a negative score if it exhibited decreased DNA methylation with aging. The outliers are strong positive and negative CpG sites and therefore given 1 or -1, respectively. Unmapped CpGs are coded as 0. All other nucleotides are given -0.00257 to ensure the maximum distance of 250 bp between any two CpGs within a segment (see below).

The maximum distance between two adjacent CpGs is determined based upon the pattern of spatial correlation of DNA methylation in the human genome. The correlation rapidly decreases to the baseline near or before ~500 bp (S10 Fig). Consequently, we used 100, 250 and 500 bp and found that the results are highly similar (e.g., S8C Fig). We present results using the maximal distance between two adjacent CpGs as 250 bp (more details are provided in the S1 Text). Under this scheme, CpGs with a stronger increase or decrease of DNA methylation are given higher absolute scores. We then used the calculated scores as templates for the maximal segment algorithm using a custom in-house script (available upon request). Among the initially identified segments, only those subsequences (‘aging segments’) that exhibited statistically significant associations with age in a linear model (FDR-corrected *q*-value < 0.05) were retained. Consequently, we identified maximal clusters of adjacent CpGs that increase DNA methylation with aging (positive segments) and those that decrease DNA methylation with aging (negative segments).

### Gene ontology, permutation test, and visualization

The DAVID 6.7 functional annotation tools [72,73] were used to examine enrichments of specific gene ontology (GO) terms. *P*-values were adjusted using the Bonferroni correction. Enrichment of aging CpGs from other datasets in our segments was performed by a permutation test as follows: if our aging segments have *n* CpGs overlapping with another dataset, we randomly choose *n* CpGs from the Illumina 27K chip and counted the number of overlaps with another data set, designated as *m*. This procedure was repeated *T* (= 100,000) times, and empirical *P*-values were calculated as: $P\sim \frac{T[m>n]}{T}$. We used R base [74] and ggplot2 [75] plotting systems to generate figures. The Gviz package was used to visualize and annotate UCSC tracks [76].

## Supporting Information

S1 Fig CpG methylation level distribution in different genomic regions (A) in blood and (B) brain. (PDF)

S2 Fig Contrasting patterns of DNA methylation change with aging for extremely hyper- and hypo-methylated CpGs. (PDF)

S3 Fig Supporting matrices for the 14 chromatin state maps of brain. (PDF)

S4 Fig The proportions of CpG sites in the whole genome belonging to each chromatin state. (PDF)

S5 Fig Distribution of regression coefficients from the linear model of aging and DNA methylation in the brain data set. (PDF)

S6 Fig DNA methylation levels across three ages in regions with fixed chromatin states in 9 different cell lines (Ernst et al. [35]). (PDF)

S7 Fig Total lengths of positive and negative aging segments in brain and blood. (PDF)

S8 Fig Overlaps between aging segments and aging CpGs identified in other studies [15,51,52]. (PDF)

S9 Fig Mean regression coefficients for mapped CpGs in different chromatin states. (PDF)

S10 Fig DNA methylation levels of neighboring CpGs are highly correlated yet decrease rapidly to the baseline near or before 500 bp. (PDF)

S1 Table Positive and negative aging segments in blood and brain data. (XLSX)

S2 Table Gene ontology enrichment of aging segments in different chromatin states in brain. (PDF)

S3 Table Genes located in common aging segments. (XLSX)

S1 Text details on defining chromatin states and identifying maximal segments (PDF)
