# Supplementary material for: Impacts of Chromatin States and Long-Range Genomic Segments on Aging and DNA Methylation
Source: PLoS One. 2015 Jun 19;10(6):e0128517. doi: 10.1371/journal.pone.0128517 (PMC4475080; doi:10.1371/journal.pone.0128517)
Supplement: S1 Fig — (PDF) [file pone.0128517.s001.pdf]

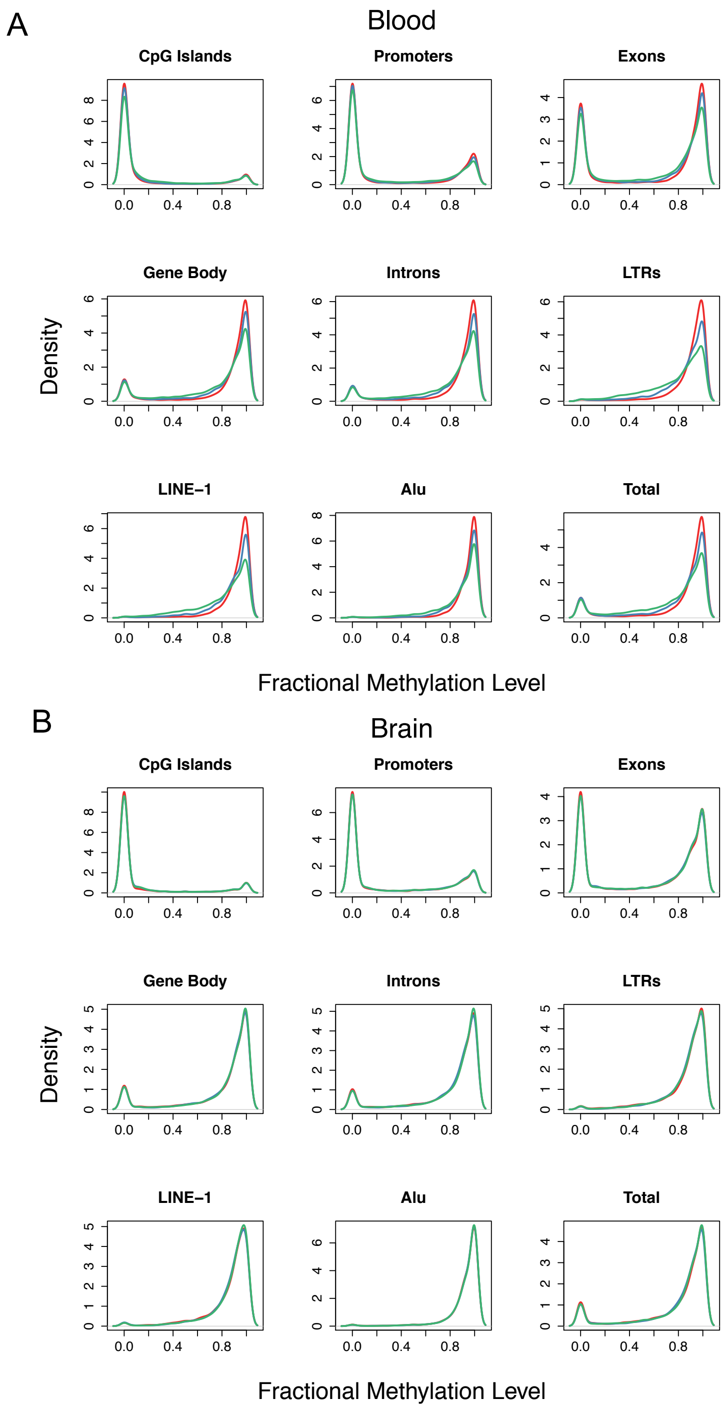

S1 Fig. CpG methylation level distribution in different genomic regions (A) in blood (B) in brain. In both tissues, data from three individuals are shown. For blood, a newborn (red), a 26-year old (blue), and a centenarian. For brain, a newborn (red), a 25-year old (blue) and an 82-year old (green).
