# Supplementary material for: Impacts of Chromatin States and Long-Range Genomic Segments on Aging and DNA Methylation
Source: PLoS One. 2015 Jun 19;10(6):e0128517. doi: 10.1371/journal.pone.0128517 (PMC4475080; doi:10.1371/journal.pone.0128517)
Supplement: S2 Fig — (PDF) [file pone.0128517.s002.pdf]

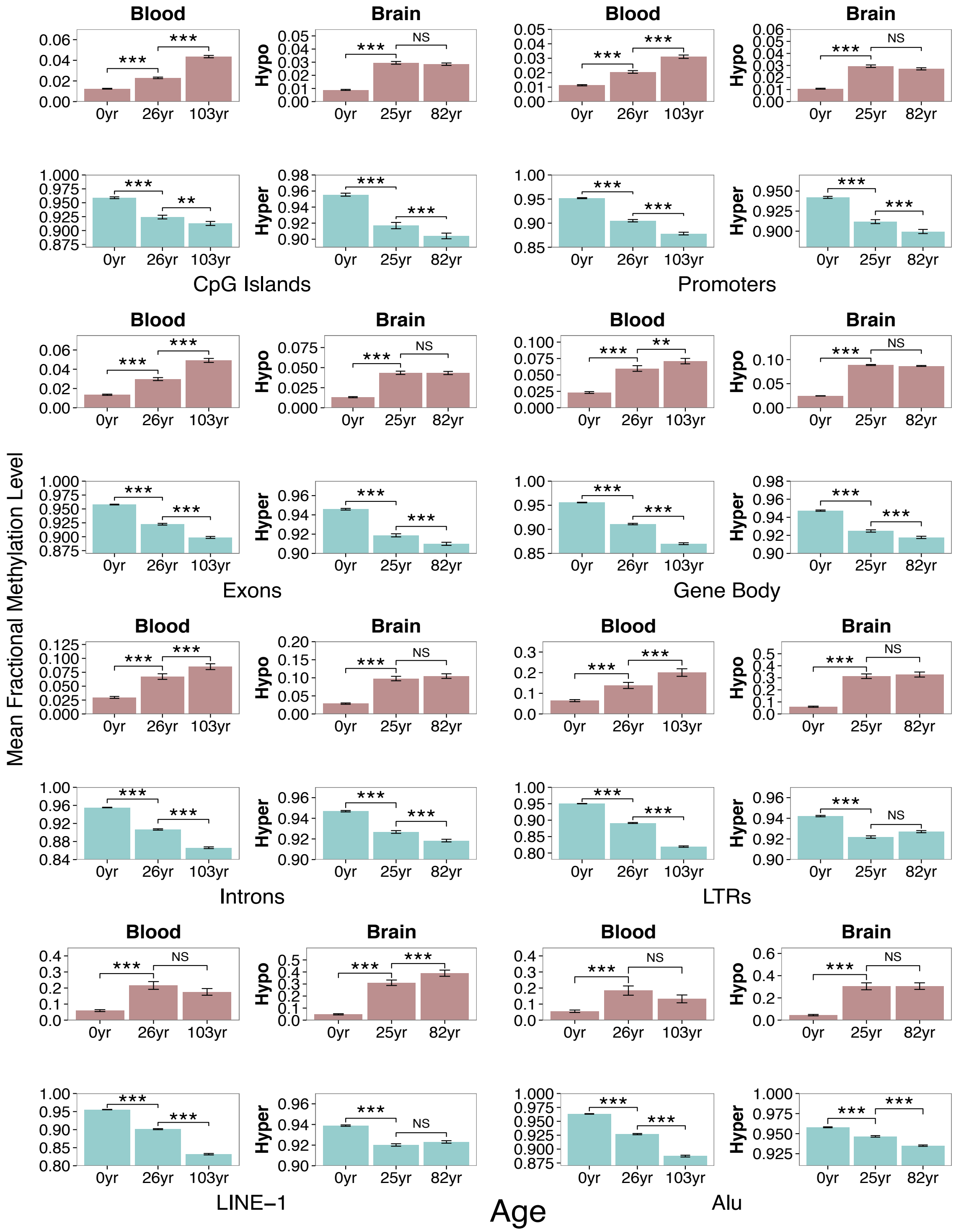

S2 Fig. Contrasting patterns of DNA methylation change with aging for extremely hyper- and hypo-methylated CpGs. Paired t-test (one-tailed) was performed for neighboring ages. P-values are marked as ***: $p < 0.001$; **: $p < 0.005$; *: $p < 0.05$; NS: not significant.
