# Supplementary material for: Impacts of Chromatin States and Long-Range Genomic Segments on Aging and DNA Methylation
Source: PLoS One. 2015 Jun 19;10(6):e0128517. doi: 10.1371/journal.pone.0128517 (PMC4475080; doi:10.1371/journal.pone.0128517)
Supplement: S3 Fig — (PDF) [file pone.0128517.s003.pdf]

A
B
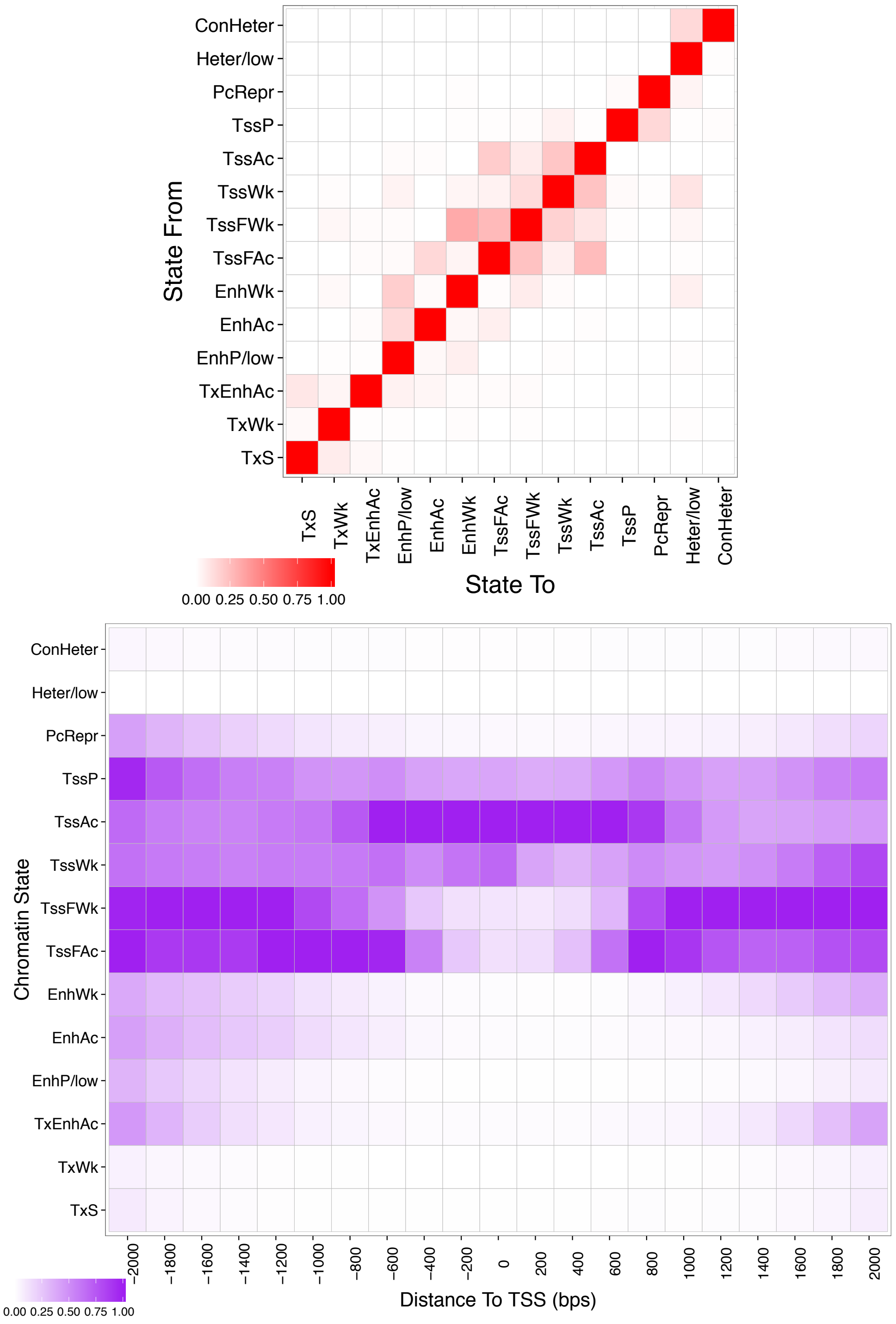

S3 Fig. Supporting matrices for the 14 chromatin states map of brain. (A) The transition probability matrix indicating the probabilities of each 200-bp (bin size) region switching from one state to another. (B) The probability matrix representing the probabilities of each 200-bp (bin size) region to be within 2kb (upstream and downstream) of the transcription start sites.
