# Supplementary material for: Impacts of Chromatin States and Long-Range Genomic Segments on Aging and DNA Methylation
Source: PLoS One. 2015 Jun 19;10(6):e0128517. doi: 10.1371/journal.pone.0128517 (PMC4475080; doi:10.1371/journal.pone.0128517)
Supplement: S4 Fig — (PDF) [file pone.0128517.s004.pdf]

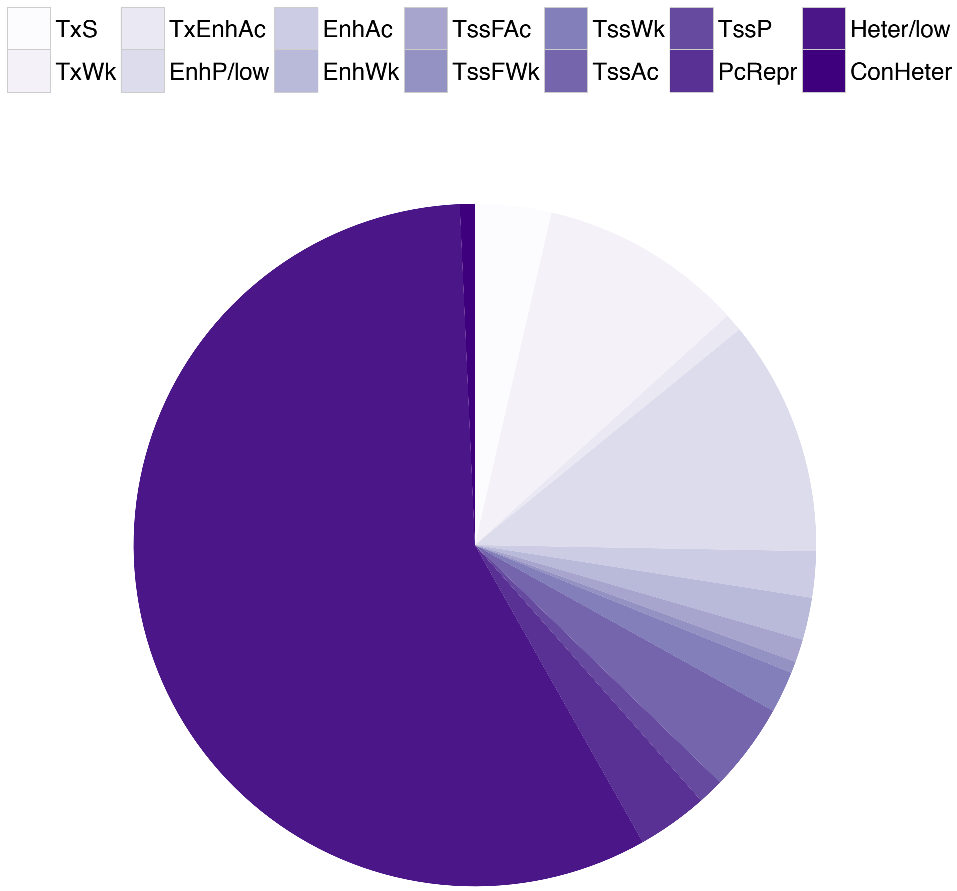

S4 Fig. The proportions of CpG sites in the whole genome belonging to each chromatin state. Heter/low makes up the highest percentage (57.48%) of the whole genome.
