# Supplementary material for: Impacts of Chromatin States and Long-Range Genomic Segments on Aging and DNA Methylation
Source: PLoS One. 2015 Jun 19;10(6):e0128517. doi: 10.1371/journal.pone.0128517 (PMC4475080; doi:10.1371/journal.pone.0128517)
Supplement: S5 Fig — (PDF) [file pone.0128517.s005.pdf]

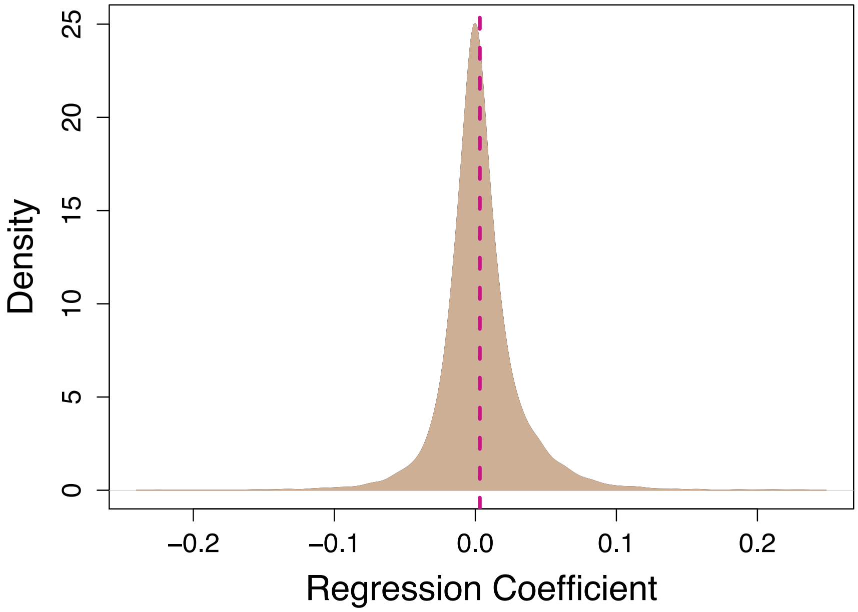

S5 Fig. Distribution of regression coefficients from the linear model of aging and DNA methylation in the brain data set. They range between -0.142 and 0.198.
