# Supplementary material for: Impacts of Chromatin States and Long-Range Genomic Segments on Aging and DNA Methylation
Source: PLoS One. 2015 Jun 19;10(6):e0128517. doi: 10.1371/journal.pone.0128517 (PMC4475080; doi:10.1371/journal.pone.0128517)
Supplement: S6 Fig — (PDF) [file pone.0128517.s006.pdf]

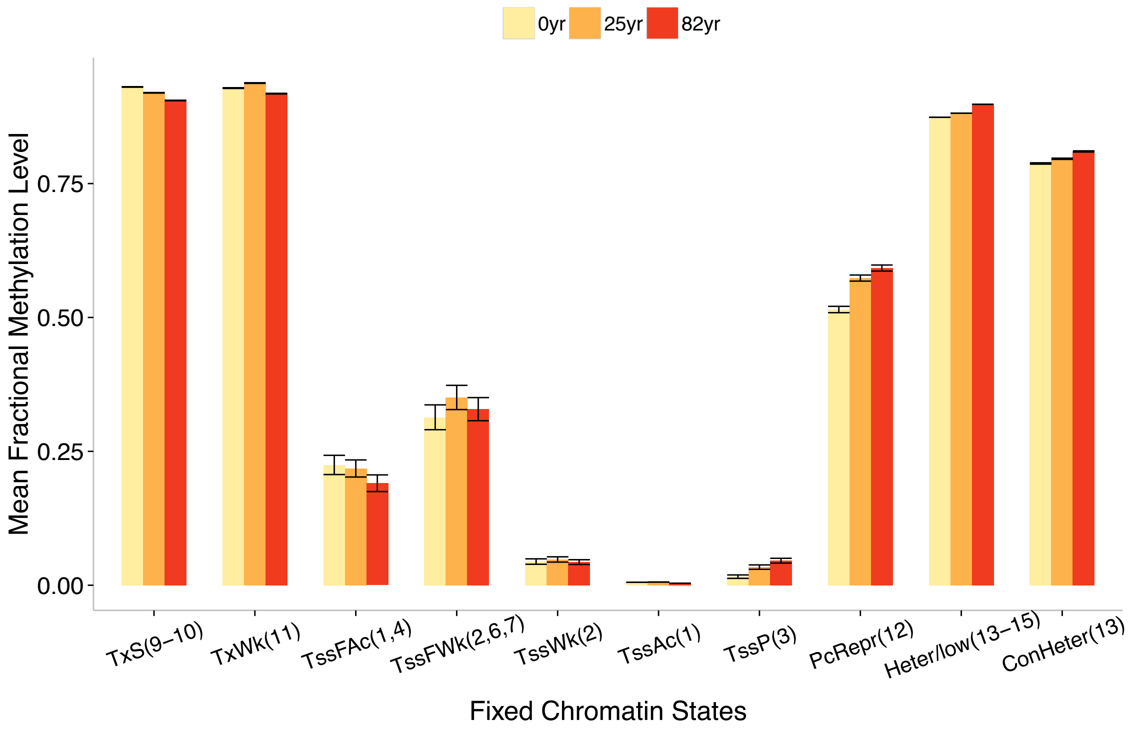

S6 Fig. DNA methylation levels across three ages in regions that have fixed chromatin states in 9 different cell lines from Ernst et al. (2011). The correspondent state numbers from Ernst et al. (2011) are shown in parentheses.
