# Supplementary figures and images for: Impacts of Chromatin States and Long-Range Genomic Segments on Aging and DNA Methylation
Source: PLoS One. 2015 Jun 19;10(6):e0128517. doi: 10.1371/journal.pone.0128517 (PMC4475080; doi:10.1371/journal.pone.0128517)

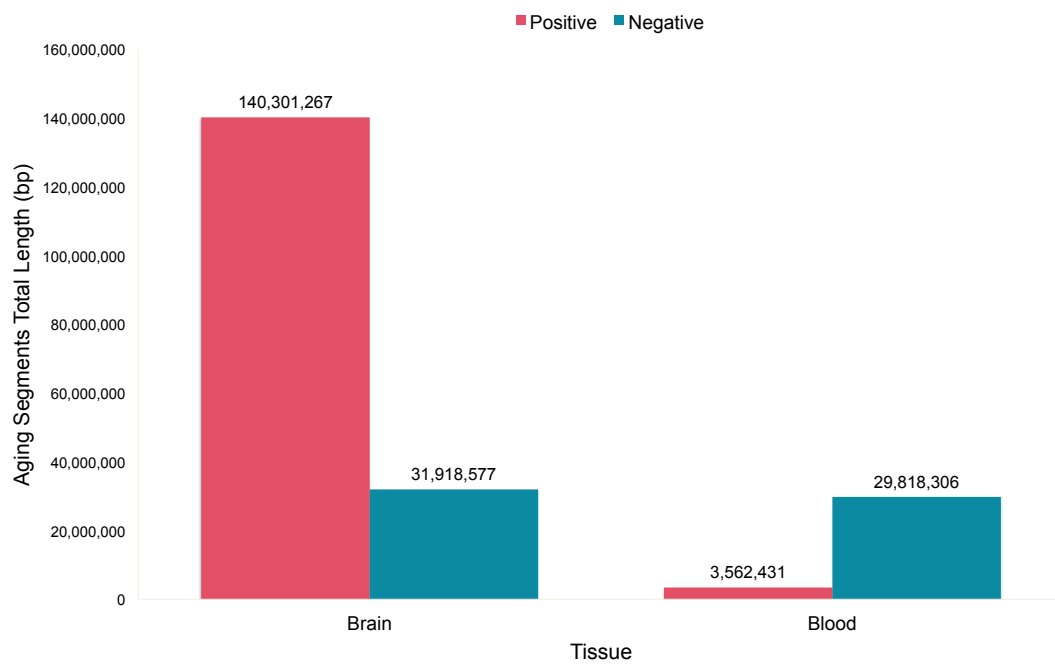

**S7 Fig.** Total lengths of positive and negative aging segments in brain and blood.

Supplement: S7 Fig — (PDF) [file pone.0128517.s007.pdf]
