# Supplementary material for: Impacts of Chromatin States and Long-Range Genomic Segments on Aging and DNA Methylation
Source: PLoS One. 2015 Jun 19;10(6):e0128517. doi: 10.1371/journal.pone.0128517 (PMC4475080; doi:10.1371/journal.pone.0128517)
Supplement: S8 Fig — (PDF) [file pone.0128517.s008.pdf]

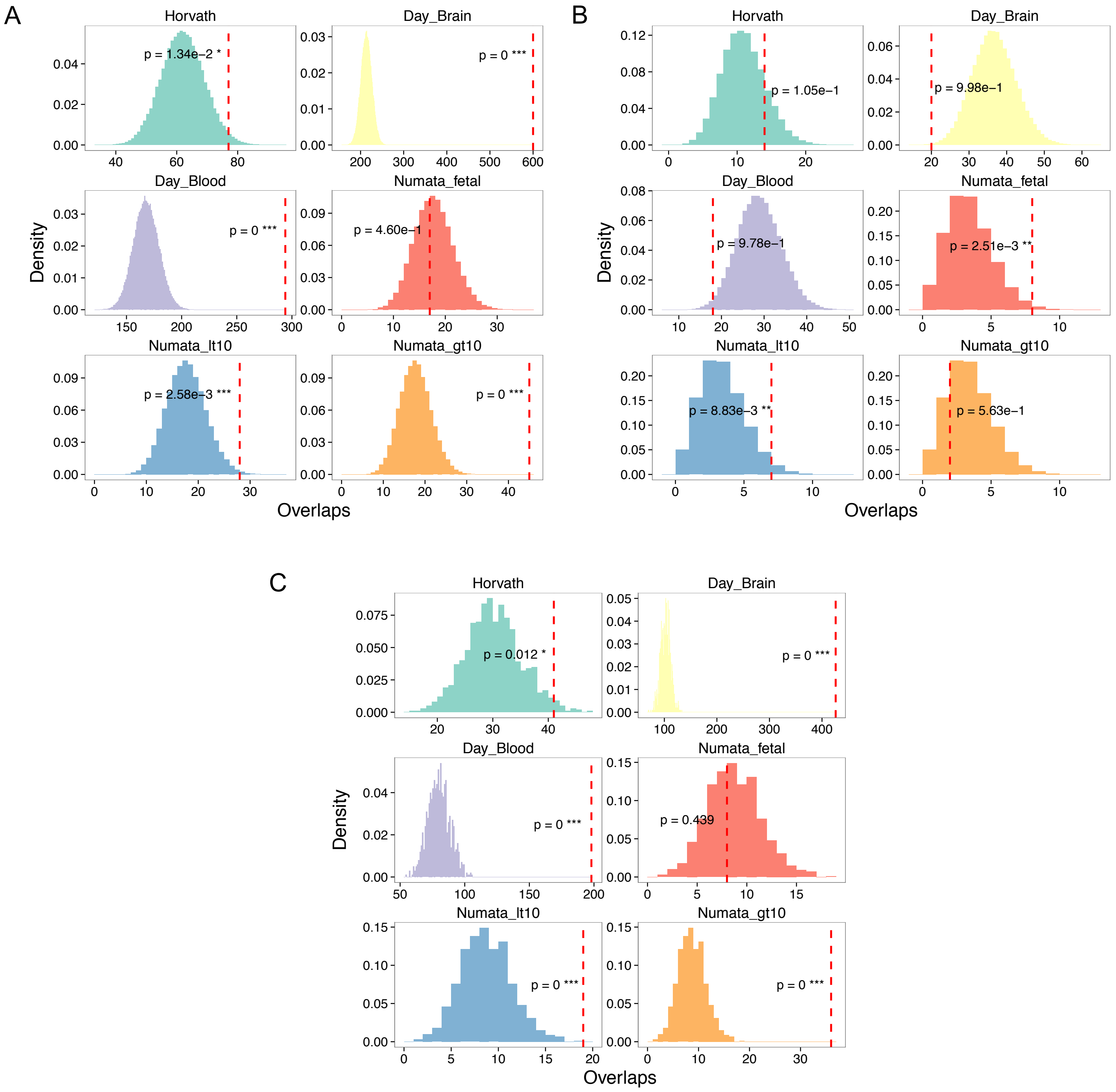

S8 Fig. Overlaps between aging segments and aging CpGs identified in other studies [1-3]. (A) Overlaps for positive aging segments. Except for fetal dataset, other aging CpGs significantly overlap with the positive aging segments. (B) Overlaps for negative aging segments. Only aging CpGs found in fetal data set or children significantly overlap with the negative aging segments. (C) Segments shared by 100bp, 250bp and 500bp cutoffs yield similarly significant overlaps.
