# Supplementary material for: Impacts of Chromatin States and Long-Range Genomic Segments on Aging and DNA Methylation
Source: PLoS One. 2015 Jun 19;10(6):e0128517. doi: 10.1371/journal.pone.0128517 (PMC4475080; doi:10.1371/journal.pone.0128517)
Supplement: S9 Fig — (PDF) [file pone.0128517.s009.pdf]

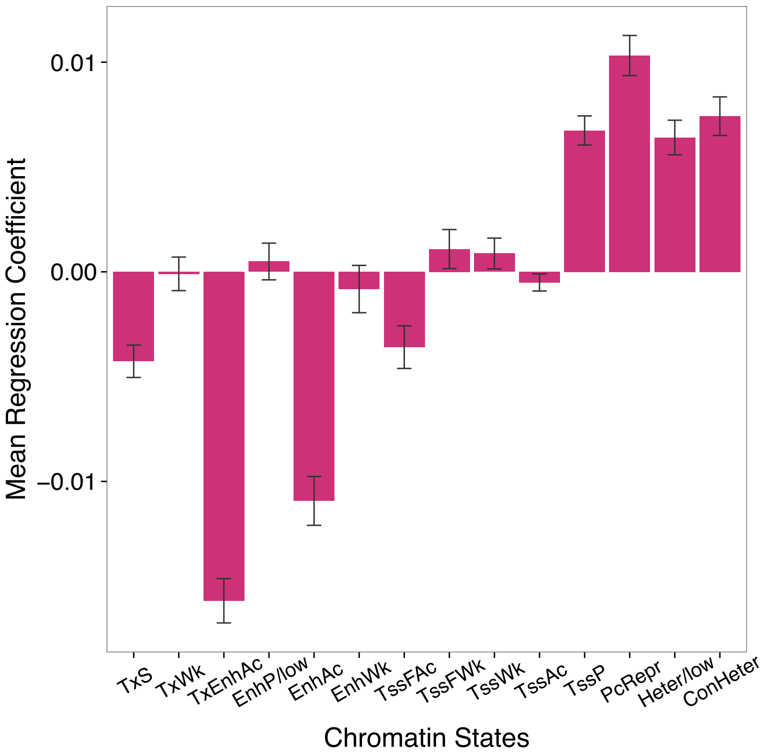

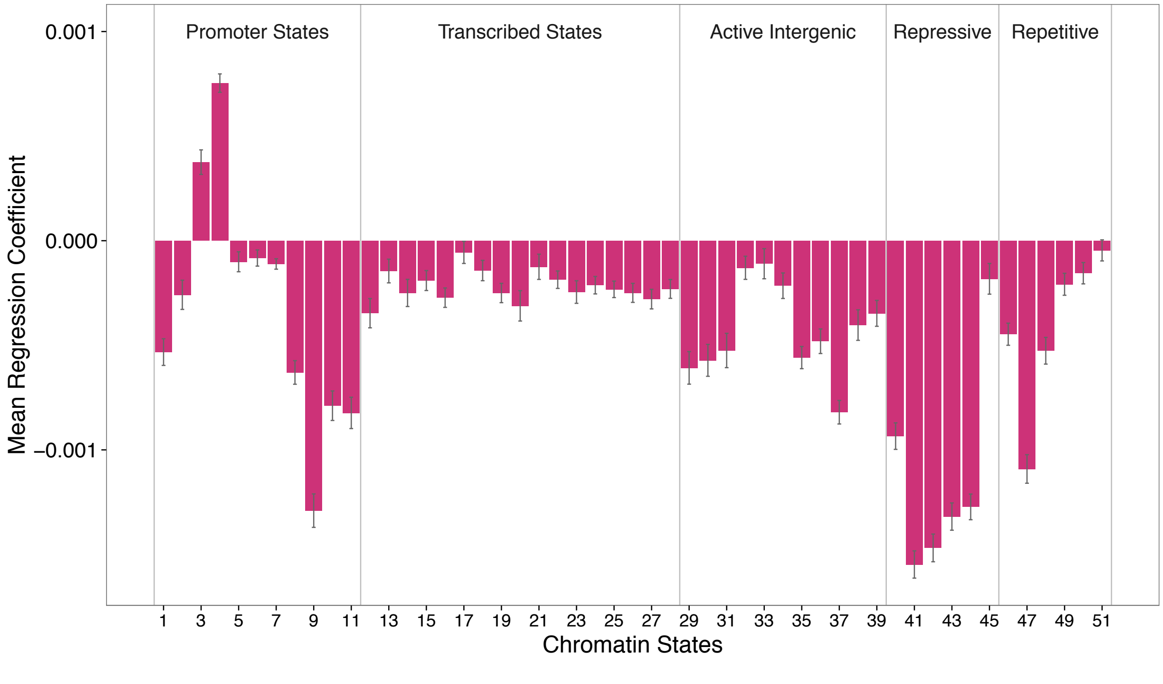

S9 Fig. Mean regression coefficients for mapped CpGs in different chromatin states. (A) Mean regression coefficients across 14 chromatin states in brain. (B) Mean regression coefficients across 51 chromatin states in blood (CD4+ T-cells, from [37]). State 3-4 (Repressed/Poised promoters) are the only states that increase methylation with aging in blood.
