# Supplementary material for: Impacts of Chromatin States and Long-Range Genomic Segments on Aging and DNA Methylation
Source: PLoS One. 2015 Jun 19;10(6):e0128517. doi: 10.1371/journal.pone.0128517 (PMC4475080; doi:10.1371/journal.pone.0128517)
Supplement: S10 Fig — (PDF) [file pone.0128517.s010.pdf]

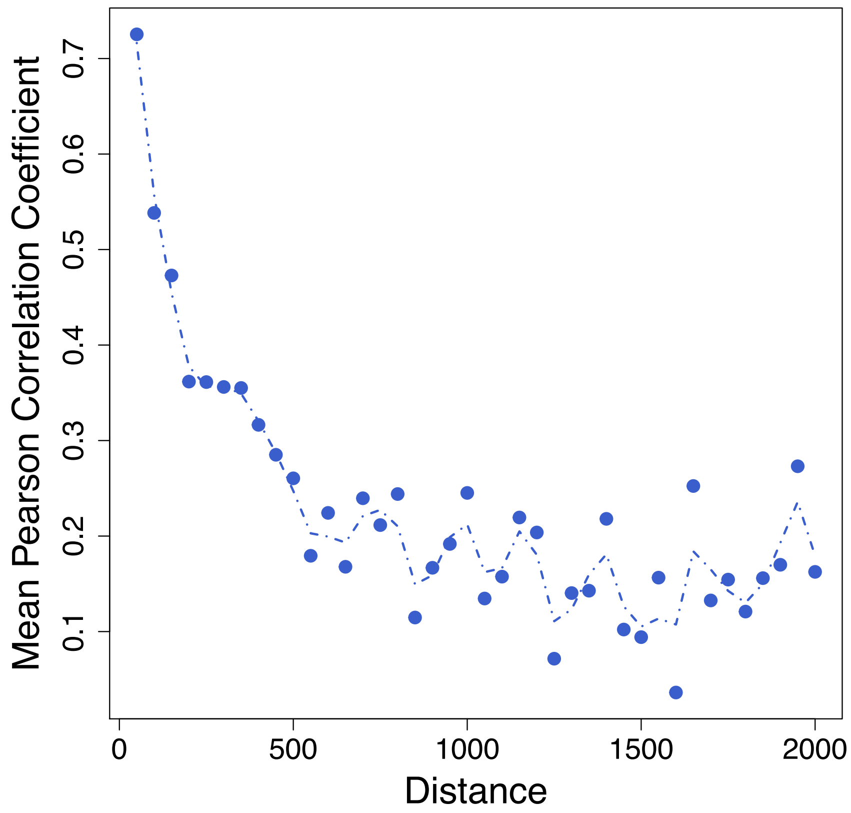

S10 Fig. DNA methylation levels of neighboring CpGs are highly correlated yet decreases rapidly to the baseline near or before 500bps.
