# Supplementary material for: Impacts of Chromatin States and Long-Range Genomic Segments on Aging and DNA Methylation
Source: PLoS One. 2015 Jun 19;10(6):e0128517. doi: 10.1371/journal.pone.0128517 (PMC4475080; doi:10.1371/journal.pone.0128517)
Supplement: S2 Table — (PDF) [file pone.0128517.s012.pdf]

S2 Table. Gene ontology enrichment of aging segments in different chromatin states in brain. Functional annotation was performed using DAVID 6.7. P-values were adjusted with Bonferroni correction.

	GO Term	Fold Enrichment	q-value
TxS (Positive Methylation Change)	Vesicle-mediated Transport	2.1	2.50E-06
	Intracellular Transport	1.9	3.80E-05
	Protein Localization	1.7	2.30E-04
	Regulation of Small GTPase Mediated Signal Transduction	2.5	3.60E-04
	Protein Transport	1.7	1.50E-03
TxS (Negative Methylation Change)	RNA Processing	2.3	2.00E-12
	mRNA Metabolic Process	2.6	2.00E-11
	RNA splicing	2.8	1.50E-10
	Chromatin Modification	2.9	3.30E-10
	mRNA Processing	2.6	1.50E-09
TxWk (Positive Methylation Change)	Chromatin Modification	2.7	5.40E-09
	Chromosome Organization	1.9	1.10E-04
	Modification-dependent Protein Catabolic Process	1.8	1.20E-04
	Modification-dependent Macromolecule Catabolic Process	1.8	1.20E-04
	Intracellular Transport	1.8	1.30E-04
TxWk (Negative Methylation Change)	Regulation of Small GTPase Mediated Signal Transduction	2.1	4.20E-10
	Protein Amino Acid Phosphorylation	1.6	1.70E-09
	Phosphorus Metabolic Process	1.5	4.10E-09
	Phosphate Metabolic Process	1.5	4.10E-09
	Phosphorylation	1.5	4.40E-07
TxEnhAc (Positive Methylation Change)	Endocytosis	4.2	9.60E-03
	Membrane Invagination	4.2	9.60E-03
	Vesicle-mediated Transport	2.7	1.00E-02
	Membrane Organization	3.1	4.90E-02
TxEnhAc (Negative Methylation Change)	Chromatin Modification	3.0	2.50E-09
	mRNA Metabolic Process	2.4	3.40E-06
	RNA Splicing	2.6	4.00E-06
	Chromatin Organization	2.4	7.60E-06
	Histone Modification	3.6	2.80E-05
TssFAc (Positive Methylation Change)	DNA Packaging	4.8	9.90E-06
	Chromatin Assembly	5.1	8.20E-05
	Protein-DNA Complex Assembly	5.0	1.60E-04
	Nucleosome Organization	4.2	2.30E-04
	Chromatin Assembly or Disassembly	5.2	2.30E-04
TssFWk (Positive Methylation Change)	M Phase	3.1	2.80E-04
	Cell Cycle Phase	2.6	3.70E-03
	Mitosis	3.4	4.40E-03
	Nuclear Division	3.4	4.40E-03
	M Phase of Mitotic Cell Cycle	3.3	5.80E-03
TssWk (Positive Methylation Change)	Nucleosome Organization	4.7	4.10E-05
	DNA Packaging	4.1	1.10E-04
	Protein-DNA Complex Assembly	4.5	1.60E-04
	Nucleosome Assembly	4.7	2.30E-04
	Chromatin Assembly	4.5	4.10E-04
TssAc (Positive Methylation Change)	DNA Packaging	4.5	4.20E-07
	Chromatin Assembly	4.9	4.90E-06
	Nucleosome Assembly	4.9	1.60E-05
	Nucleosome Organization	4.6	1.80E-05
	Protein-DNA Complex Assembly	4.5	6.70E-05
TssP (Positive Methylation Change)	Regulation of Transcription, DNA-dependent	2.4	2.40E-30
	Regulation of RNA Metabolic Process	2.4	4.50E-30
	Embryonic Morphogenesis	5.0	3.60E-24
	Pattern Specification Process	5.2	1.00E-22
	Regionalization	6.1	1.10E-22
PcRepr (Positive Methylation Change)	Pattern Specification Process	4.2	2.10E-36
	Regionalization	4.8	3.00E-35
	Embryonic Morphogenesis	3.8	4.60E-33
	Anterior/posterior Pattern Formation	5.3	1.90E-30
	Skeletal System Development	3.5	9.00E-28
