# Supplementary material for: Impacts of Chromatin States and Long-Range Genomic Segments on Aging and DNA Methylation
Source: PLoS One. 2015 Jun 19;10(6):e0128517. doi: 10.1371/journal.pone.0128517 (PMC4475080; doi:10.1371/journal.pone.0128517)
Supplement: S1 Text — (PDF) [file pone.0128517.s014.pdf]

S1 Text

Defining Chromatin States

We defined chromatin states based on the emission matrix (Figure 2A), transition matrix (S3. Fig.) and fold enrichment for RefSeq TSS (S3. Fig.) similarly to Ernst et al. 2011 [1] and Kasowski et al. [2]. Details of each state are provided below.

State1 (TxS): H3K36me3 is the signature for strong transcription [3-6].
State2 (TxWk): Same as above, but the signal for H3K36me3 is weaker.
State3 (TxEnhAc): With the strong transcription mark H3K36me3 and active enhancer marks H3K27ac and H3K4me1 [7,8], this state is annotated as enhancers that reside in transcribed regions. This does not necessarily mean these enhancers regulate the genes they are in, but also could be possibly involved in regulation of other genes [9-11].
State4 (EnhP/low): This state has low signals of all states. Based on the transition matrix, it has highest probabilities to transit to other enhancer states, and is hence considered as poised/weak enhancers.
State5 (EnhAc): Strong signals of both H3K27ac and H3K4me1 are indications of typical strong enhancers [7,8,12-16].
State6 (EnhWk): Same as above, but with weaker signals for both H3K27ac and H3K4me1 [8,17].
State7 (TssFAc): This state has strong signals for both H3K4me3 (enriched in promoters) and H3K4me1 (enriched in enhancers) and active histone modification H3K27ac. Based on Fig. S3B, this state is flanking transcription state sites and are hence defined as 5' flanking regions. This state might be composed of both active promoters and active proximal enhancers [13,14,18].
State8 (TssFWk): Same as above, but with weaker signal for H3K27ac [8,17,19,20].
State9 (TssWk): With H3K4me3 and weak H3K27ac signal, this state represents weak promoters [19,20].
State10 (TssAc): Active promoters harbor both strong H3K4me3 and H3K27ac signals [18,21-24].
State11 (TssP): Bivalent histone marks H3K27me3 and H3K4me3 and weak signals for H3K27ac are indications of poised promoters [25,26].
State12 (PcRepr): The repressive mark H3K27me3 alone defines polycomb complexes target regions [27-30].

State13 (Heter/low): Heterochromatin regions are difficult to sequence, and thus this region has extremely low signals for any histone modifications.
State14 (ConHeter): Constitutive heterochromatin harbors the repressive chromatin mark H3K9me3 [31].

Identifying Aging Segments

We used a maximal scoring subsequence algorithm [32] to define aging segments. This approach aims to find all non-overlapping and continuous subsequences with maximal local scores. For all cytosines in mapped CpGs, *t*-statistics from the above regression model were used as pre-scores. Since outliers influence unfairly for normalization, we first exclude the outliers that are not in this range:

$$\left(Q1 - 1.5 \times IQR, Q3 + 1.5 \times IQR\right)$$

To normalize the pre-scores to [-1, 1] scale, for the rest of the pre-scores, we did a normalization using the formula:

$$score = \frac{pre\_score - \min(pre\_score)}{\max(pre\_score) - \min(pre\_score)}$$

The outliers are strong positive and negative CpG sites, and were therefore given 1 or -1. All unmapped cytosines and guanines in all CpG sites were given 0. All other nucleotides were given score -0.00257 to ensure maximal 250bp between any two CpG sites within a segment. We then used the calculated scores as templates for the maximal segment algorithm (using a custom-written Perl software) to identify sub-sequences with local maximal scores. The same linear regression method was adopted as pervious described. Only those subsequences ('aging segments') that fit FDR-corrected q-value < 0.05 were kept. The same process was repeated for identifying positive and negative aging segments, respectively.

1. Ernst J, Kheradpour P, Mikkelsen TS, Shoresh N, Ward LD, Epstein CB, et al. (2011) Mapping and analysis of chromatin state dynamics in nine human cell types. Nature 473: 43-49.
2. Kasowski M, Kyriazopoulou-Panagiotopoulou S, Grubert F, Zaugg JB, Kundaje A, Liu Y, et al. (2013) Extensive variation in chromatin states across humans. Science 342: 750-752.

3. Bannister AJ, Schneider R, Myers FA, Thorne AW, Crane-Robinson C, Kouzarides T (2005) Spatial distribution of di- and tri-methyl lysine 36 of histone H3 at active genes. J Biol Chem 280: 17732-17736.
4. Barski A, Cuddapah S, Cui K, Roh TY, Schones DE, Wang Z, et al. (2007) High-resolution profiling of histone methylations in the human genome. Cell 129: 823-837.
5. Edmunds JW, Mahadevan LC, Clayton AL (2008) Dynamic histone H3 methylation during gene induction: HYPB/Setd2 mediates all H3K36 trimethylation. EMBO J 27: 406-420.
6. Kolasinska-Zwierz P, Down T, Latorre I, Liu T, Liu XS, Ahringer J (2009) Differential chromatin marking of introns and expressed exons by H3K36me3. Nat Genet 41: 376-381.
7. Bogdanovic O, Fernandez-Minan A, Tena JJ, de la Calle-Mustienes E, Hidalgo C, van Kruysbergen I, et al. (2012) Dynamics of enhancer chromatin signatures mark the transition from pluripotency to cell specification during embryogenesis. Genome Res 22: 2043-2053.
8. Creyghton MP, Cheng AW, Welstead GG, Kooistra T, Carey BW, Steine EJ, et al. (2010) Histone H3K27ac separates active from poised enhancers and predicts developmental state. Proc Natl Acad Sci U S A 107: 21931-21936.
9. Eichenlaub MP, Ettwiller L (2011) De novo genesis of enhancers in vertebrates. PLoS Biol 9: e1001188.
10. Dong X, Navratilova P, Fredman D, Drivenes O, Becker TS, Lenhard B (2010) Exonic remnants of whole-genome duplication reveal cis-regulatory function of coding exons. Nucleic Acids Res 38: 1071-1085.
11. Birnbaum RY, Clowney EJ, Agamy O, Kim MJ, Zhao J, Yamanaka T, et al. (2012) Coding exons function as tissue-specific enhancers of nearby genes. Genome Res 22: 1059-1068.
12. Shlyueva D, Stampfel G, Stark A (2014) Transcriptional enhancers: from properties to genome-wide predictions. Nat Rev Genet 15: 272-286.
13. Pekowska A, Benoukraf T, Zacarias-Cabeza J, Belhocine M, Koch F, Holota H, et al. (2011) H3K4 tri-methylation provides an epigenetic signature of active enhancers. EMBO J 30: 4198-4210.
14. Heintzman ND, Hon GC, Hawkins RD, Kheradpour P, Stark A, Harp LF, et al. (2009) Histone modifications at human enhancers reflect global cell-type-specific gene expression. Nature 459: 108-112.
15. Zentner GE, Tesar PJ, Scacheri PC (2011) Epigenetic signatures distinguish multiple classes of enhancers with distinct cellular functions. Genome Res 21: 1273-1283.
16. Rada-Iglesias A, Bajpai R, Swigut T, Brugmann SA, Flynn RA, Wysocka J (2011) A unique chromatin signature uncovers early developmental enhancers in humans. Nature 470: 279-283.
17. Cotney J, Leng J, Oh S, DeMare LE, Reilly SK, Gerstein MB, et al. (2012) Chromatin state signatures associated with tissue-specific gene expression and enhancer activity in the embryonic limb. Genome Res 22: 1069-1080.

18. Heintzman ND, Stuart RK, Hon G, Fu YT, Ching CW, Hawkins RD, et al. (2007) Distinct and predictive chromatin signatures of transcriptional promoters and enhancers in the human genome. Nature Genetics 39: 311-318.
19. Wang Z, Zang C, Rosenfeld JA, Schones DE, Barski A, Cuddapah S, et al. (2008) Combinatorial patterns of histone acetylations and methylations in the human genome. Nat Genet 40: 897-903.
20. Karlic R, Chung HR, Lasserre J, Vlahovicek K, Vingron M (2010) Histone modification levels are predictive for gene expression. Proc Natl Acad Sci U S A 107: 2926-2931.
21. Schneider R, Bannister AJ, Myers FA, Thorne AW, Crane-Robinson C, Kouzarides T (2004) Histone H3 lysine 4 methylation patterns in higher eukaryotic genes. Nat Cell Biol 6: 73-77.
22. Santos-Rosa H, Schneider R, Bannister AJ, Sherriff J, Bernstein BE, Emre NC, et al. (2002) Active genes are tri-methylated at K4 of histone H3. Nature 419: 407-411.
23. Bernstein BE, Kamal M, Lindblad-Toh K, Bekiranov S, Bailey DK, Huebert DJ, et al. (2005) Genomic maps and comparative analysis of histone modifications in human and mouse. Cell 120: 169-181.
24. Bernstein BE, Humphrey EL, Erlich RL, Schneider R, Bouman P, Liu JS, et al. (2002) Methylation of histone H3 Lys 4 in coding regions of active genes. Proc Natl Acad Sci U S A 99: 8695-8700.
25. Voigt P, Tee WW, Reinberg D (2013) A double take on bivalent promoters. Genes Dev 27: 1318-1338.
26. Bernstein BE, Mikkelsen TS, Xie X, Kamal M, Huebert DJ, Cuff J, et al. (2006) A bivalent chromatin structure marks key developmental genes in embryonic stem cells. Cell 125: 315-326.
27. Simon JA, Kingston RE (2009) Mechanisms of polycomb gene silencing: knowns and unknowns. Nat Rev Mol Cell Biol 10: 697-708.
28. Min J, Zhang Y, Xu RM (2003) Structural basis for specific binding of Polycomb chromodomain to histone H3 methylated at Lys 27. Genes Dev 17: 1823-1828.
29. Fischle W, Wang Y, Jacobs SA, Kim Y, Allis CD, Khorasanizadeh S (2003) Molecular basis for the discrimination of repressive methyl-lysine marks in histone H3 by Polycomb and HP1 chromodomains. Genes Dev 17: 1870-1881.
30. Margueron R, Reinberg D (2011) The Polycomb complex PRC2 and its mark in life. Nature 469: 343-349.
31. Puschendorf M, Terranova R, Boutsma E, Mao XH, Isono KI, Brykczynska U, et al. (2008) PRC1 and Suv39h specify parental asymmetry at constitutive heterochromatin in early mouse embryos. Nature Genetics 40: 411-420.
32. Ruzzo WL, Tompa M (1999) A linear algorithm for finding all maximal scoring subsequences. 7th Intl conf Intelligent Systems for Molecular Biology, Heidelberg, Germany: 234-241.
